# Twenty-Four-Month Efficacy of Ureteroureterostomy Combined With Unilateral Nephrostomy Following Radical Cystectomy

**DOI:** 10.7759/cureus.30478

**Published:** 2022-10-19

**Authors:** Christos Papadimitriou, Wilfried Martin, Athanasios E Dellis, Charalampos Deliveliotis, Iraklis Mitsogiannis

**Affiliations:** 1 Department of Urology, St. Antonius Hospital Gronau, Gronau, DEU; 2 Department of Urology, Agaplesion General Hospital Hagen, Hagen, DEU; 3 2nd Department of Surgery, National and Kapodistrian University of Athens, Athens, GRC; 4 2nd Department of Urology, Sismanoglio Hospital, National and Kapodistrian University of Athens, Athens, GRC

**Keywords:** ureteroureterostomy, urinary diversion, pyonephrosis, renal function, cystectomy

## Abstract

Introduction

The purpose of this study was to evaluate the 24-month outcomes of ureteroureterostomy combined with unilateral nephrostomy following radical cystectomy in patients with muscle-invasive bladder cancer (BC).

Materials and methods

This single-center study with prospectively collected data with retrospective data analysis was carried out between December 2018 and November 2021 and enrolled 36 patients, who underwent radical cystectomy combined with ureteroureterostomy and unilateral nephrostomy. Regular renal function assessment was carried out using serum creatinine and estimated glomerular filtration rate (eGFR), and postoperative complications, endoscopic, ultrasound, and other radiological study findings were evaluated. The follow-up of the patients was carried out over a period of 24 months.

Results

After completion of the 24-month follow-up, the renal function proved to be slightly improved (mean serum creatinine and eGFR values of 1.38±0.72 mg/dL and 55.9±21.87 mL/min) compared to the first-year results (1.41±0.54 mg/dL and 52.10±19.64 mL/min). However, this improvement is statistically not significant (p=0.44, p=0.30).

The 24-month follow-up imaging findings remained stable in 97.22% of patients compared to the first-year results, with preservation of bilateral ureteric dilatation and grade 1 dilatation of the non-drained kidney. No case of acute pyelonephritis was recorded after the completion of the second year of follow-up, in comparison to the eight patients (22.22%) of the 12-month follow-up, who suffered acute pyelonephritis.

After completing of the 24-month follow-up, one patient was excluded from further analysis, due to the placement of a second permanent percutaneous nephrostomy in the non-drained kidney, due to ureteroureterostomy stenosis with consecutive hydronephrosis in the contralateral kidney and acute renal failure. No case of anastomotic leak was observed.

Conclusions

The function of the ureteroureterostomy combined with unilateral nephrostomy is proven to be a safe method of urinary diversion (UD) at 24 months, with minimal and easily manageable complications. Only one case of stenosis of the ureteroureterostomy with consecutive acute renal failure due to hydronephrosis in the non-drained kidney was observed. The renal function remained stable.

## Introduction

Ureteroureterostomy combined with unilateral nephrostomy is a non-continent urinary diversion that is widely unknown and poorly studied. There are reports that Boari first discussed this specific type of urinary diversion (UD) as early as the year 1984 [1]. Ileal conduit, first described in 1911 by Zaayer and further developed in 1950 by Bricker, is nowadays the most preferred form of non-continent urinary diversion [2,3]. In elderly patients, the use of types of non-continent UD such as ileal conduit and ureterocutaneostomy is preferred, but ureterocutaneostomy after radical cystectomy is considered a regular procedure to avoid complications associated with the use of intestinal segments without compromising the oncological result [4].

A detailed description of the surgical procedure and its peri and postoperative course is presented in our previous publication [5]. We previously reported that ureteroureterostomy combined with unilateral nephrostomy is a safe and effective method of UD following radical cystectomy for muscle-invasive bladder cancer (BC) with easily manageable complications within the first 12 months after surgery [5]. We are currently presenting the 24-month outcomes after ureteroureterostomy combined with unilateral nephrostomy following radical cystectomy for muscle-invasive BC.

## Materials and methods

The demographic data and size of the cohort of our study, which was conducted in the Department of Urology, Agaplesion General Hospital Hagen, Germany, remained unchanged compared to our previous publication [5], as presented in Table 1.

**Table 1:** Demographic data of the patients included in the study.

Sex of the patients	
Male	26
Female	10
Total	36
Age of the patients	
Minimum	56
Maximum	90
Average	77.44
SD	8.58

The overall preoperative health condition of 86.11% of the cohort was rated ≥3 based on the fitness classification system of the American Society of Anesthesiologists (ASA). In addition, 33 out of a total of 36 patients have high comorbidity with an age-adjusted Charlson Comorbidity Index score ≥6. The mean operation time was 166 min.

The current study included patients who underwent radical cystectomy with ureteroureterostomy combined with unilateral nephrostomy with muscle-invasive BC with stage T2-4b N0/N1 M0/M+ without osseous metastases, being at high surgical or anesthesiologic risk and/or low life expectancy. Patients having a solitary kidney, stricture of the upper third of the ureter, bilateral staghorn calculi, retroperitoneal fibrosis, history of previous ureterolysis, or being on hemodialysis or in metastatic bone disease were excluded from the study.

Our criterion for absolute success was the survival of the patient without anastomosis complication requiring open surgical revision, with a non-fatal complication of anastomosis and estimated glomerular filtration rate (eGFR) reduction of less than 25%. Qualified success was defined as the survival of the patient without anastomosis complications requiring open surgical revision as well as without fatal complications of anastomosis.

The diagnostic criteria of acute pyelonephritis were a positive urine culture (>10^5^ colony-forming units) and clinical signs of either frank pain or tenderness with fever (38.5˚C), which was observed in the absence of another indication for fever.

The scheduled postoperative follow-up visits in our clinic continued uninterrupted during the second year of our study during 15th, 18th, 21st, and 24th postoperative months. The nephrostomy tube was regularly exchanged every six weeks, under fluoroscopic guidance.

During the visits, we continued the clinical examination, laboratory studies, and sonographic and radiological studies. Serum creatinine and estimated glomerular filtration rate (eGFR) continued to be used as markers of renal function. The tenets of the Declaration of Helsinki were fully respected. The study was approved by the local institutional review board.

Statistical Package for the Social Sciences (SPSS) version 22 (Armonk, NY: IBM Corp.) was used to perform the statistical calculations. A power analysis using G*Power 3 (Kiel, Germany: Franz Faul, University of Kiel) to test the difference from constant in one sample using an effect size of d=0.80 and an alpha of 0.05 showed that 19 participants were required to achieve a power of 0.95 [6]. Independent sample t-tests were used for the group comparison. Statistical significance was set at 5% (p=0.05).

## Results

The size of the cohort remained the same compared to the 12-month follow-up data. The research continued for 26 men and 10 women, i.e., a total of 36 patients, with a mean age of 77.4±8.6 years. Patients were followed for a mean of 26.67±7.5 months. We present our data at the 24-month follow-up. One more patient was excluded in the 21st month from further analysis and was added to the two excluded patients of the first year of follow-up, due to placement of a second percutaneous nephrostomy in the non-drained kidney, due to ureteroureterostomy stenosis with consecutive hydronephrosis in the contralateral kidney and acute renal failure. Pyonephrosis was discovered incidentally during pelvic puncture. The administration of intravenous antibiotics based on a previous antibiogram, as well as the drainage of the obstructed kidney, led to prevention of inflammation and normalization of the renal function. Computed tomography findings revealed extraluminal compression of the anastomosis and ureters on both sides of the ureteroureterostomy at a distance of 1 cm, due to local disease recurrence. Due to the imaging findings, it was decided in palliative setting to permanently drain both kidneys through percutaneous nephrostomies. At the end of the 24-month follow-up, 8.33% of patients experienced some ureteroureterostomy complication.

In our previous article unilateral dilatation of the contralateral renal pelvis was postoperatively observed in 13 patients (36.1%) over the 12-month-follow-up period [3]. In the present study, no further patient beyond the aforementioned 13 patients developed dilatation of the non-drained kidney. Also, at the end of the 24-month follow-up, there was a reduction in patients with unilateral kidney dilatation from 13 to 11 patients. This is due to the exclusion of one patient from the further study, due to stenosis of the anastomosis with placement of a second nephrostomy as a permanent solution. One further patient of the above cohort was removed due to death. The cause of death was the progression of the disease. The bilateral ureteric dilatation persisted in all patients during the entire second year.

**Figure 1:**
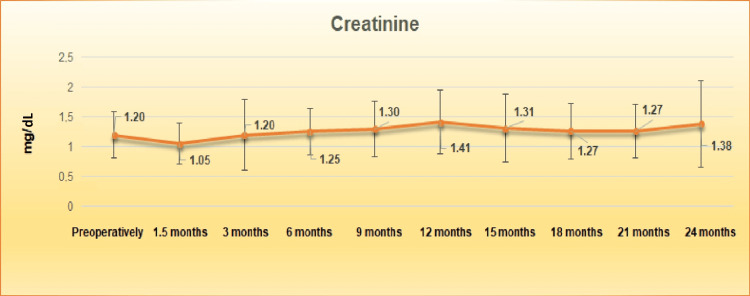
Graph of the mean creatinine curve (mg/dL) with the standard deviation up to 24 months.

**Figure 2:**
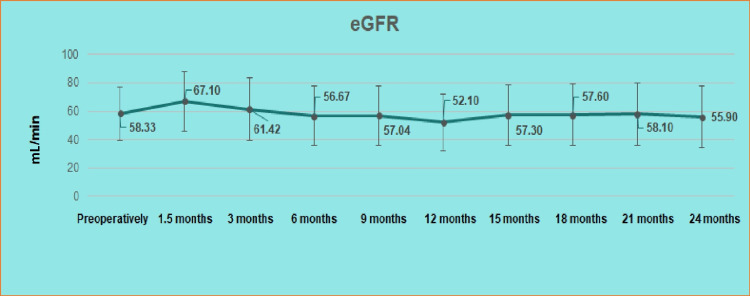
Graph of the mean eGFR (mL/min) with the standard deviation up to 24 months. eGFR: estimated glomerular filtration rate

Preoperatively the serum creatinine and the eGFR fluctuated between 0.52 and 2.73 mg/dL and 20.4 and 119.2 mL/min, respectively; the mean serum creatinine and eGFR were 1.20±0.39 mg/dL and 58.33±18.88 mL/min, respectively. The average serum creatinine and eGFR after six-month follow-up was 1.25±0.39 mg/dL (p=0.30) and 56.67±21.05 mL/min (p=0.38), respectively, and after 12 months postoperatively 1.41±0.54 mg/dL (p=0.05) and 52.10±19.64 mL/min (p=0.13), respectively. The average serum creatinine and eGFR after 24 months (1.38±0.72 mg/dL {p=0.44} and 55.90±21.87 mL/min {p=0.30}, respectively) were not significantly improved than the serum creatinine and eGFR values measured at 12 months (Figures 1, 2).

**Figure 3:**
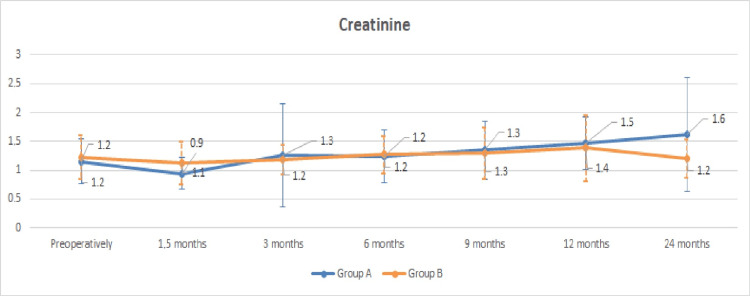
Graph of the mean creatinine curve (mg/dL) in patients with and without unilateral pelvic dilatation. Group A: group of patients with postoperative unilateral pelvic dilatation in the non-drained kidney. Group B: group of patients without postoperative pelvic dilatation in the non-drained kidney.

**Figure 4:**
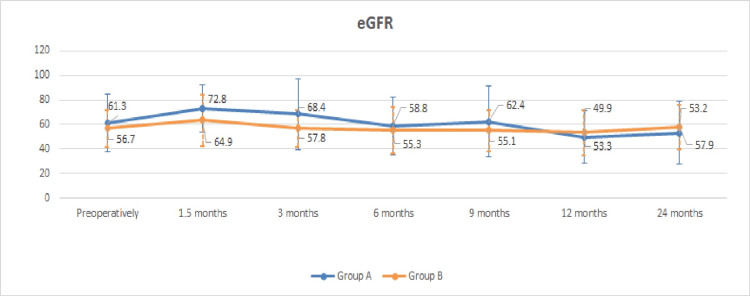
Graph of the mean eGFR curve (mL/min) in patients with and without unilateral pelvic dilatation. Group A: group of patients with postoperative unilateral pelvic dilatation in the non-drained kidney. Group B: group of patients without postoperative pelvic dilatation in the non-drained kidney. eGFR: estimated glomerular filtration rate

The course of renal function proves to be stable in the current study in the group of patients with postoperative unilateral pelvic dilatation in the non-drained kidney; mean serum creatinine increased from 1.15±0.40 mg/dL preoperatively to 1.47±0.46 mg/dL after 12 months (p=0.09) and 1.61±0.98 mg/dL after 24 months (p=0.11) (Figure 3), whereas eGFR decreased from a preoperative 61.27±23.83 mL/min to 49.90±21.21 mL/min (p=0.18) at the 12-month follow-up and stabilized at 53.24±25.90 mL/min at the 24-month follow-up (p=0.27) (Figure 4).

At the end of the second year, there was no statistically significant difference in the rate of change in renal function between patients with and without renal dilatation in the non-drained kidney. Complications at 24 months related to nephrostomy or ureterostomy are limited (Table 2).

**Table 2:** Nephrostomy- and ureteroureterostomy-related complications.

Complications	Time of the complication	Type of complication			Number of patients
Nephrostomy	13-24 months	Dislodgement			5
		Obstruction			3
		Hematuria after change			2
		Bacteriuria			22
Ureteroureterostomy	13-24 months		Stenosis	1	
			Ureterohydronephrosis grade 4 of the contralateral kidney	1	
			Pyonephrosis	1	
			Acute renal failure	1	
			Placement of a second permanent percutaneous nephrostomy at the contralateral site	1	
			Ureterohydronephrosis grade 1 of the contralateral kidney	11	

Obstruction of the anastomosis due to stenosis with consecutive acute renal failure and pyonephrosis in the contralateral kidney was noticed in one case and was treated with the insertion of a contralateral percutaneous nephrostomy tube and administration of intravenous antibiotic therapy. Anastomotic leak was not observed. The nephrostomy complications were minor without the need for intervention or hospitalization.

**Figure 5:**
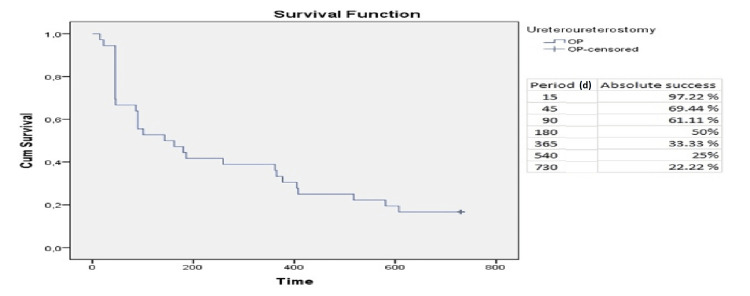
Absolute success - Kaplan-Meier curve at 730 days. d: days

**Figure 6:**
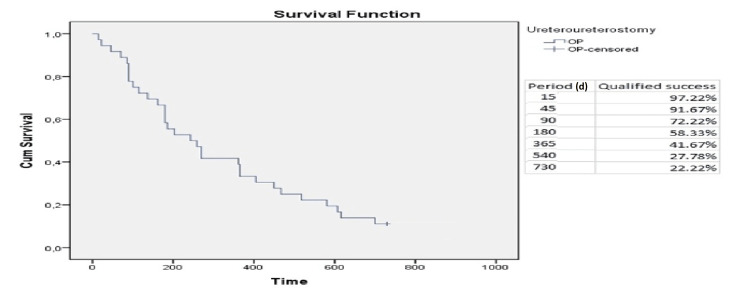
Relative success - Kaplan-Meier curve at 730 days. d: days

Summarizing the above results, the cumulative probability of absolute success was 50%, 33.3%, and 22.2% at six months, 12 months, and 24 months, respectively (Figure 5). Moreover, we observed a cumulative probability of qualified success in 58.3%, 41.7%, and 22.2% at six months, 12 months, and 24 months, respectively (Figure 6).

## Discussion

The ureteroureterostomy is statistically recorded as safe UD. Primary and secondary complications were recorded in only 8.33% of patients. Minimally invasive management without general anesthesia was sufficient to successfully manage these complications.

A careful analysis of the data records that only 22.22% of the sample meets both success criteria. We have to take into consideration that these statistical results are due to the reduction of the sample to half of the preoperative cohort. The causes of death of the above-mentioned patients are not anastomosis related but mainly the progression of the disease and natural causes due to the advanced age of the sample. Lemiński et al. and Aziz et al. studies confirm our findings, as older age is documented as an independent factor for increased all-cause mortality among patients with muscle-invasive BC undergoing radical cystectomy [7,8].

Jancke et al. reported in a sample of eight patients, using a non-stenting anastomosis technic, two cases of ureteroureterostomy failure (stricture, leakage) with no need for open surgical revision [9]. Better results are presented in our study. Out of the three patients with an anastomosis complication, one case is not evaluated as a direct complication of the ureteroureterostomy, as the anastomosis was injured during sigmoidectomy in the context of intestinal ileus treatment due to extensive local progression of the urothelial carcinoma. The preoperative computed tomography revealed an absence of dilatation of the renal pelvicalyceal system without pathological findings in the anastomosis area and proves that the anastomosis complication was caused due to surgical manipulation. No case of anastomosis complications required open surgical revision, as well as no case of fatal complication of anastomosis, was recorded. The incidence of ureteral anastomotic leakage or stricture is 3.6% and 10%, respectively, requiring open surgical revision in 7.9% and 14.2%, respectively, in patients with an ileal conduit [10]. Only 5.55% of our patients developed anastomotic complications. This percentage is lower compared to the 8.33% incidence of ureterocutaneous anastomotic stricture in ureterocutaneostomy patients, as presented by Kızılay et al. [11].

Siddiqui and Izawa reported a 20.3% rate of gastrointestinal complications, 2.1% incidence of stomal stenosis as well as 14% parastomal hernia after radical cystectomy with ileal conduit formation [12]. The absence of such complications in our study confirms our previous belief that it is a safe UD, especially for elderly patients.

We consider that the average operative time (166 min) is short and directly comparable to the fastest ureterocutaneostomy surgeries (131-461 min) and shorter than the ileum conduit formation (215-510 min) [13]. Possibly this factor also contributes to the smooth postoperative course.

The course of the renal function of the living patients at the 24-month follow-up is considered satisfactory, as a slight improvement was observed in the mean value of serum creatinine and eGFR compared to the 12 months, 2.12% and 6.76%, respectively. Compared to preoperative renal markers, there is a slight deterioration in mean creatinine and eGFR, 13.04% and 4.16%, respectively. The above-mentioned data, as well as the fact, that pelvic dilatation in the non-drained kidney is recorded as a factor that does not negatively affect renal function, confirm the postoperative unremarkable mercaptoacetyltriglycine (MAG-3) renography findings after ureteroureterostomy of Jancke et al. [9], which confirm the experimental data on the ureteroureterostomy function due to the presence of ureteric antiperistalsis of Melick et al. [14]. In their retrospective study of 169 patients with bladder cancer who underwent radical cystectomy and UD (cutaneous ureterostomy, ileal conduit, neobladder substitution), Nishikawa et al. demonstrated that the incidence of renal deterioration was independent of the type of UD with a mean reduction of eGFR by 19.68% (from 69.6 to 55.9 mL/min/1.73 m^2^) during the mean observation period of 103.8 months. These data strengthen our data that ureteroureterostomy maintains renal function over time at satisfactory levels (4.16% deterioration of eGFR at 24 months) [15].

During the 12-month follow-up, at the end of 24 months, all patients had asymptomatic bacteriuria. New episodes of acute pyelonephritis were not recorded during the second year of the study, and the patient population remained stable throughout the second postoperative year (22.22%). We believe that a key role in the prevention of new episodes of acute pyelonephritis was played by familiarizing patients with the correct use and recognition of complications of the nephrostomy system (tube, urine collection bag) and prompt attendance in our urologic clinic in case of nephrostomy malfunction. A mean incidence of 25.4% for acute pyelonephritis was reported by Nishikawa et al. for three different UD (cutaneous ureterostomy, ileal conduit, neobladder) and specifically in 33.3%, 27.5%, and 20.7%, respectively. Non-continent UD is reported to be more prone to upper urinary tract infections [15].

Patients with pyonephrosis increased in total by three patients after the completion of the 24-month follow-up. This incident was an accidental finding in the context of the placement of a second percutaneous nephrostomy in the non-drained kidney, due to ureteroureterostomy stenosis with consecutive hydronephrosis in the contralateral kidney and acute renal failure. Preoperatively both clinical and laboratory findings did not suspect inflammation. Due to the manipulation, intravenous antibiotics based on a previous antibiogram were administered to prevent postinterventional inflammation.

Creating a urostomy or a continent UD (neobladder, pouch) in an obese patient may be particularly challenging as it is accompanied by an increased possibility of complications [16]. The ureteroureterostomy is prepared in the same way regardless of the size of the abdominal wall, just as the placement of the percutaneous nephrostomy in the prone position is usually without technical difficulties.

Considering the aforementioned complications, we consider that the majority of our patients experience a non-complicated postoperative course. Isolated cases of nephrostomy obstruction or dislocation were promptly and successfully treated by changing the nephrostomy tube.

Limitations of our study remain the small number of patients and the reduction of the sample to half of the preoperative cohort in comparison to that after 24-month follow-up. Further studies are needed to evaluate the long-term efficacy and safety of ureteroureterostomy in combination with unilateral nephrostomy following radical cystectomy.

## Conclusions

Summarizing the above results ureteroureterostomy combined with unilateral nephrostomy following radical cystectomy remains a safe and effective method of UD for frail elderly patients with muscle-invasive BC. Our experience surgically, with easy anastomosis preparation independently of the patient, and clinically, with simple postoperative care and follow-up of these vulnerable patients combined with minimal complications and preservation of renal function, strengthens the argument that this type of UC can be established as an option in daily practice. Especially in cases of severe obesity in vulnerable elderly patients, ureteroureterostomy is regarded as a very useful alternative surgical procedure. Larger, longer-term studies are needed to confirm these results.
